# The effectiveness of COVID-19 vaccination in preventing hospitalisation and mortality: A nationwide cross-sectional study in Iran

**DOI:** 10.7189/jogh.14.05026

**Published:** 2024-09-27

**Authors:** Mahya Razimoghadam, Rajabali Daroudi, Mehdi Yaseri

**Affiliations:** 1 Department of Health Management, Policy and Economics, School of Public Health, Tehran University of Medical Sciences, Tehran, Iran; 2 National Center for Health Insurance Research, Tehran, Iran; 3 Department of Epidemiology and Biostatistics, School of Public Health, Tehran University of Medical Sciences, Tehran, Iran

## Abstract

**Background:**

The pandemic of the coronavirus disease 2019 (COVID-19) led to a global health crisis, prompting widespread vaccination efforts to reduce severe outcomes. In this study, we assessed the impact of mass COVID-19 vaccination on hospitalisation and mortality rates in Iran, where over 83% of the vaccinated population received inactivated virus vaccines.

**Methods:**

Using retrospective, cross-sectional analysis, we examined data from the Iran Health Insurance Organisation, covering 41 million individuals from 20 February 2020 to 20 March 2022. We analysed hospital records from 956 Iranian hospitals, focusing on inpatient stays, short-term hospitalisations, and emergency department visits. Study outcomes included COVID-19 hospital admissions and associated mortality. We used negative binomial regression to compare hospital admission rates between periods, while we used a poison regression model with a log link to assess mortality risks before and after vaccination.

**Results:**

Among 806 076 hospital admissions, 57 599 deaths were recorded. COVID-19 hospitalisations increased with age, and women had slightly higher admission rates than men. Advanced age and male sex correlated with higher mortality rates. Hospital admissions rose to 1178.66 per million population per month post-vaccination compared to 459.78 pre-vaccination. The incidence rate ratio was 2.09 (95% confidence interval (CI) = 1.90–2.32, *P* < 0.001), mainly due to the Delta variant. In contrast, post-vaccination mortality rates decreased from 111.33 to 51.66 per 1000 admissions per month. Post-vaccination, COVID-19 mortality significantly decreased, with a relative risk being 0.61 (95% CI = 0.60–0.62, *P* < 0.001) across all age groups and sexes.

**Conclusions:**

The Delta variant increased hospital admissions among vaccinated individuals, but widespread vaccination significantly reduced COVID-19-related mortality.

Over three years have passed since the coronavirus disease 2019 (COVID-19) outbreak, caused by the severe acute respiratory syndrome coronavirus 2 (SARS-CoV-2), leading to a global pandemic. As of July 2024, the World Health Organization (WHO) has reported more than 767 million confirmed cases and 6 947 192 deaths due to COVID-19 [1]. Various health protocols, such as lockdowns, social distancing, face masks, and the use of hand sanitisers, were implemented in response to the COVID-19 outbreak. While these measures have been instrumental in curbing the spread of the virus, they did not fully resolve the global health emergency. Vaccination emerged as a pivotal solution to mitigate the severe impacts of COVID-19 and has played a central role in the efforts to end the worldwide health crisis. Numerous studies have consistently shown that vaccination significantly decreases the risk of COVID-19 infection, related hospitalisations, and deaths [2–27].

The Iranian officials formally declared the first documented instance of SARS-CoV-2 infection on 19 February 2020. In Iran, COVID-19 had a severe impact, becoming the leading cause of emergency department visits over two years [28]. As of May 2024, the SARS-CoV-2 virus has impacted over 7.6 million individuals in Iran, leading to more than 146 837 reported fatalities [29]. Iran began its COVID-19 vaccination campaign in February 2021, prioritising health care professionals. By July 2023, 77.61% of Iran’s population had received at least one dose of a COVID-19 vaccine [1]. The Iranian populace received a range of vaccines, such as Sputnik V (viral vector), Oxford/AstraZeneca (viral vector), Covaxin (inactivated), Sinopharm (inactivated), and several domestically produced vaccines. Approximately 83% of the administered doses consisted of the inactivated virus vaccine Sinopharm [30,31]. The remaining vaccines were predominantly AstraZeneca and Sputnik V, each accounting for less than 9% of the total doses imported to Iran [31].

Previous research within Iran has indicated the efficacy of the COVID-19 vaccine in reducing hospital admissions and mortality rates in a few provinces. For instance, a cohort study conducted in Fars province revealed that vaccinated individuals experienced a reduction in hospitalisations by at least 67% and a decrease in mortality rates by 85%. This research further highlighted that vaccinated individuals exhibited a lower incidence rate of COVID-19 infections [32]. Furthermore, findings from a test-negative case-control study in Gilan province illustrated that post-vaccination, there was a decline in both hospitalisation and mortality rates for almost all vaccines available in Iran. It was also observed that hospitalisations and deaths diminished progressively post-immunisation [33]. According to previous research, the Sinopharm and Oxford/AstraZeneca vaccines provided effective immunity against SARS-CoV-2 in a limited population of Iranians [34]. One study demonstrated that the Sinopharm vaccine is 94.4% effective in children aged 12–17 years who have received at least two doses [35]. Prior studies in Iran mainly focused on individual hospitals, specific provinces, or particular age groups. This study is the first to explore the effects of extensive COVID-19 vaccination on hospitalisations and mortality rates nationwide in Iran. In contrast to previous studies that concentrated on the immunogenicity and reactogenicity of specific vaccines, in this research, we assessed the impact of vaccination on a nationwide population. Specifically, we investigated the effectiveness of different vaccine types, mostly inactivated and viral vector vaccines.

## METHODS

### Study setting

In this retrospective, cross-sectional study, we used data from the Iran Health Insurance Organisation (IHIO) covering 42 million individuals. IHIO is one of Iran’s primary insurance companies. The insurance is inclusive since it has five funds with different plans for various populations. These plans benefit a wide range of groups in a country, including civil servants, rural citizens, urban citizens, and immigrants. The IHIO has contracts with 956 hospitals across all Iranian provinces, representing nearly 88% of Iran’s hospitals. Using data collected from these hospitals, we conducted a comprehensive nationwide analysis.

We sourced the data from the electronic health record system of the Ministry of Health and Medical Education of Iran. IHIO receives these claims data every month, and the data used for this research were sourced from the National Centre for Health Insurance research. The Information Technology and Statistics Management Centre at the Ministry of Health and Medical Education of Iran has developed standardised protocols for data exchange. In collaboration with the IHIO’s Department of Statistics and Information Technology, this centre ensures the standardisation of medical records and maintains high data quality.

### Study outcomes and variables

**Figure 1:**
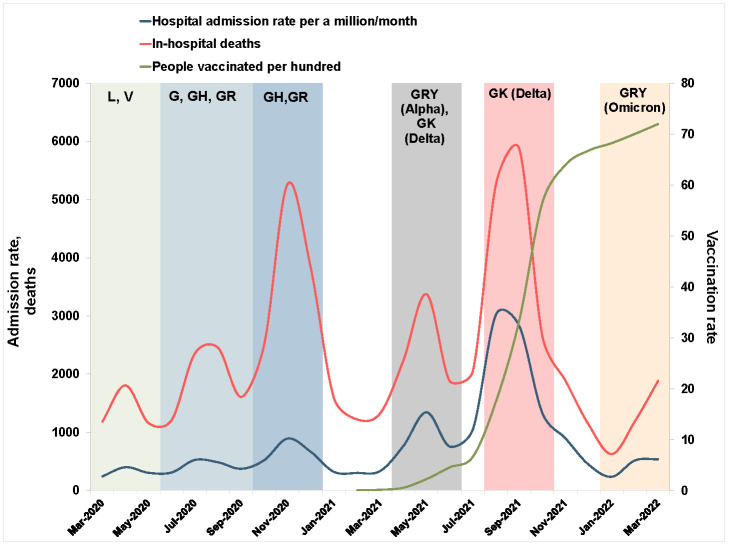
COVID-19 hospital admissions, in-hospital deaths, and people vaccinated per 100 with at least one dose during the pandemic, with SARS-CoV-2 clades indicated for each time frame.

The primary outcome of this study pertains to COVID-19-related hospital admissions. We employed the 10th revision of the International Classification of Diseases to categorise hospital health conditions. In Iran, the codes U07.1 and U07.2 identify COVID-19 patients. Our study incorporated patients who were primarily diagnosed with COVID-19. There were no missing data, and all medical records were thoroughly examined. Patients aged <12 years were excluded based on the claim data. Hospital admissions data are presented in terms of age, sex, type of hospital, registration method, and discharge status. Five age categories are recognised: 12–17 years, 18–44 years, 45–64 years, 65–74 years, and ≥75 years. Sex, determined at birth, is classified into two categories – female and male. The other variable is the insurance fund that represents the specified insurance plan for each population category. This includes civil servants, rural citizens, urban citizens, and immigrants. The registration types include inpatient, short-term hospitalisation, and emergency department (ED). Inpatient hospitalisations last at least one night, while short-term hospitalisations last less than six hours. We characterised the ED visit as a patient being registered in the ED, remaining under observation, and receiving services without being officially admitted as an inpatient. We did not incorporate outpatient claims data. The secondary outcome of the study focuses on in-hospital mortality related to COVID-19. We extracted mortality data from discharge status in hospital records. We carried out mortality analyses with stratifications based on age and sex. The genome surveillance data and vaccination information during the COVID-19 pandemic in Iran are presented in **Figure 1**. Genome surveillance and vaccination information were obtained from previous studies and official statistical reports from Iran [36–39].

### Study periods

The study spans from 20 February 2020 to 20 March 2022. It’s divided into two distinct phases – pre-vaccination and post-vaccination. The pre-vaccination period encompassed the time frame between 20 February 2020 and 20 April 2021, which served as a baseline for measuring the impact of vaccination. The post-vaccination period extended from 21 April 2021 to 20 March 2022, during which the effects of vaccination were evaluated and compared against the pre-vaccination data. Initially, health care workers were the primary recipients of the COVID-19 vaccines in Iran. Subsequent vaccinations were rolled out based on age group prioritisation. The commencement of the general COVID-19 vaccination phase within this study was marked by the point when 1% of the Iranian population had received at least one dose of an approved COVID-19 vaccine.

We conducted age-specific analyses to account for the unique vaccination timelines associated with each age group. In accordance with an official announcement made by the Ministry of Health and Medical Education, specific age groups were granted the opportunity to register for COVID-19 vaccination through the designated website. Upon registration, individuals were given details regarding their vaccination schedules and appointment location. This study aimed to determine the onset of immunity for each age group, considering one month following the authorisation for online registration within their respective age category.

The one-month timeframe was selected based on the predominant use of the Sinopharm inactivated virus vaccine in Iran, where the second dose is typically administered 21–28 days after the initial dose [35,40–42]. This period was chosen to allow adequate time for the administration of the second dose and the subsequent development of an immune response. This timeframe aligns with the established immunisation schedule for Sinopharm and similar vaccines, which generally achieve significant immunity within a few weeks following the second dose [43–45]. For instance, individuals aged ≥75 years were slated for vaccination in late April 2021. Thus, their pre-vaccination period was from 20 February 2020 to 21 May 2021, and the post-vaccination stretch was from 22 May 2021 to 20 March 2022. Vaccination dates were set as 23 July 2021 for those aged 65–74 years, 23 August 2021 for those aged 45–64, 23 September 2021 for individuals aged 18–44, and 23 October 2021 for those aged 12–17.

### Statistical analysis

A hospital admission rate was calculated per million population per month by dividing the number of admissions by the number of IHIO members during each period. Age- and sex-specific admission rates were reported. We calculated the mortality risk by subtracting the number of cases discharged by death status from the number of hospital admissions during that period. We categorised mortality by age and sex and reported it per 1000-monthly admissions. As a count variable with an overdispersion, we analysed the admissions with a negative binomial regression, a proper model for these data [46]. The incidence rate ratio (IRR) was estimated using negative binomial regression to compare admission rates per million population per month in aggregated data. We considered the pre-vaccination period as the baseline. An exposure variable was the total number of members of IHIO during different time periods. We adjusted IRRs for age, sex and insurance funds. To estimate IRR by age and sex, we entered IHIO members in the same age and sex groups as exposure variables.

We used a Poisson regression with a log link to compare post-vaccination with pre-vaccination mortality risks. The relative risk (RR) was deduced based on individual mortality binary data using a Poisson regression with no offset added. We adjusted RR for age, sex, and insurance funds. We conducted statistical analyses using Stata, version 17 (StataCorp LLC, College Station, Texas, USA). IRR and RR are presented with 95% confidence intervals (CIs). A *P*-value of 0.05 is deemed statistically significant.

## RESULTS

The COVID-19 hospital admission rate was estimated at 776.09 per million from 20 February 2020 to 20 March 2022. During this period, there were 57 599 in-hospital deaths due to COVID-19. Significant peaks in COVID-19 deaths were observed during the third and fifth waves of the pandemic in Iran, with death numbers reaching 5266 and 5877, respectively (**Figure 1**). During the fifth wave, when the Delta variant was dominant in Iran, hospital admissions peaked at 3024.22 per million population per month.

### Hospital admissions

From 20 February 2020 to 20 March 2022, 806 076 hospital visits in Iran were recorded, with the primary diagnosis being COVID-19. The age group with the fewest hospital admissions was children aged 12–17 years, whereas the age group of 45–64 years witnessed the highest admissions. The rate of hospital admissions due to COVID-19 increased with age. Of the total admissions, 53% were women. Of the total hospital admissions in this study, 554 510 were inpatient admissions, and 234 666 were admissions to the hospital ED. In terms of percentages, inpatient admissions comprised 68%, ED admissions 29%, and short-term inpatient admissions roughly 2% of the total admissions (**Table 1**).

**Table 1:** Characteristics of COVID-19 hospital admissions and the hospital admission rate per a million population/mo among members of the Iran Health Insurance Organisation

Items	Total, n (%)	Admission rate (per million/1000 population)		*P*-value*
		**Pre-vaccination**	**Post-vaccination**	
Hospital admissions	806 076 (100)	459.78	1178.66	<0.001
Age in years				
*12–17*	5967 (0.74)	63.89	76.42	0.046
*18–44*	216 281 (26.83)	466.20	411.91	<0.001
*45–64*	298 721 (37.06)	1345.26	1843.06	<0.001
*65–74*	139 795 (17.34)	2436.10	4024.50	<0.001
*≥75*	145 312 (18.03)	3779.45	4756.80	<0.001
Sex				
*Female*	424 071 (52.61)	472.71	1308.08	<0.001
*Male*	382 005 (47.39)	447.54	1056.34	<0.001
Insurance fund				
*Rural citizens*	289 693 (35.94)	350.45	852.29	<0.001
*Urban citizens*	207 969 (25.80)	317.06	940.53	<0.001
*Civil servants*	218 864 (27.15)	954.68	2603.21	<0.001
*Immigrants*	3280 (0.41)	966.00	2145.13	<0.001
*Other*	86 270 (10.70)	1301.29	2475.19	<0.001
Record status				
*Inpatient*	554 510 (68.79)	396.70	708.49	<0.001
*Short-time hospitalisation*	14 569 (1.81)	2.33	29.55	<0.001
*Emergency department*	234 666 (29.11)	59.43	437.83	<0.001
*Unspecified*	2331 (0.29)	1.81	2.80	<0.001

*All *P*-values are statistically significant (*P* < 0.05).

In the pre-vaccination period, during the initial year of the COVID-19 outbreak in Iran, hospital admission rates stood at 459.78 per million population per month. This rate surged to 1178.66 in the post-vaccination phase during the outbreak’s second year. The increase was observed across all age groups of >45 years and both sexes. Specifically, the admission rate for the age group of 45–64 years rose from 1345.26 to 1843.06 per million population per month. Similarly, those aged 65–74 years and those aged ≥75 experienced an increase from 2436.10 and 3779.45 to 4024.50 and 4756.80, respectively. For females, the COVID-19 hospital admission rate escalated from 472.71 to 1308.08 per million population per month post-vaccination. For males, it rose from 447.54 to 1056.34 post-vaccination. Hospital admissions increased significantly across various insurance fund categories. Among rural citizens, the admission rate rose markedly from 350.45 per million population per month before vaccination to 852.29 after vaccination. Urban citizens experienced a similar trend, with admissions increasing from 317.06 per million population per month to 940.53. Civil servants saw a substantial rise in hospital admissions, from 954.68 per million population per month pre-vaccination to 2603.21 post-vaccination. Immigrants also experienced a significant increase, with admissions growing from 966.00 per million population per month to 2145.13 following vaccination. Before vaccination, there were 340.04 recoveries from COVID-19 per million population per month, a rate that soared to 997.62 post-vaccination.

**Figure 2:**
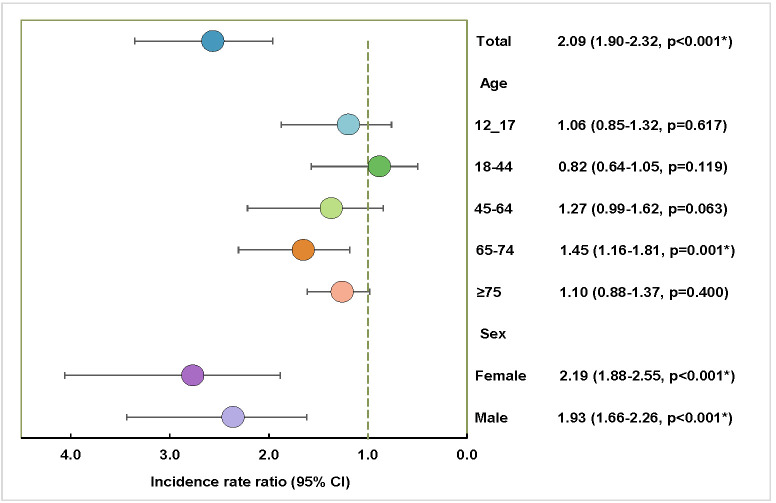
Incidence rate ratio of COVID-19 hospital admissions after vaccination compared to the pre-vaccination period as the baseline in Iran. *Significant *P*-values (*P* < 0.05).

The adjusted IRR for hospital admissions post-vaccination compared to pre-vaccination was 2.09 (95% CI = 1.90–2.32, *P* < 0.001). Assessing age-specific vaccination timelines, the age groups of 12–17, 45–64, and ≥75 years showed an uptick in post-vaccination hospital admission rates, but the changes were not statistically significant. However, the age group of 65–74 years witnessed a rise in post-vaccination hospital admissions compared to pre-vaccination (IRR = 1.45; 95% CI = 1.16–1.81, *P* = 0.001). Both female and male populations exhibited increased hospital admissions post-vaccination (**Figure 2**).

### In-hospital mortality

There were 1984 deaths per month due to COVID-19 at the hospital before vaccination and 2530 after vaccination. The highest number of in-hospital deaths associated with COVID-19 occurred in older adults (aged ≥75 years), with 25 560 reported deaths. Based on a reference age group of 12–17 years, all age groups had higher mortality rates. An adjusted odds ratio (OR) of 1.29 indicates that men have a higher risk of COVID-19 mortality than women. Between record status, inpatient cases have higher mortality than short-term inpatients and ED patients (**Table 2**).

**Table 2:** Number of COVID-19 in-hospital deaths and associated odds ratios

	Total, n (%)	Unadjusted OR (95% CI)	*P*-value	Adjusted OR (95% CI)	*P*-value
**Mortality**	57 599 (100)				
Pre-vaccination	29 768 (52.00)	ref		ref	
Post-vaccination	27 831 (48.00)	0.43 (0.43–44)	<0.001	0.72 (0.70–0.73)	<0.001*
**Age in years**					
12–17	114 (0.00)	ref		ref	
18–44	4293 (7.00)	1.04 (0.86–1.25)	0.684	1.14 (0.94–1.37)	0.183
45–64	14 009 (24.00)	2.53 (2.10–3.04)	<0.001*	2.62 (2.17–3.16)	<0.001*
65–74	13 623 (24.00)	5.54 (4.60–6.68)	<0.001*	5.22 (4.33–6.29)	<0.001*
≥75	25 560 (44.00)	10.96 (9.10–13.20)	<0.001*	9.29 (7.70–11.19)	<0.001*
**Sex**					
Female	26 036 (45.00)	ref		ref	
Male	31 563 (55.00)	1.38 (1.35–1.40)	<0.001*	1.29 (1.27–1.32)	<0.001*
**Insurance fund**					
Rural citizens	19 253 (33.00)	ref		ref	
Urban citizens	13 271 (23.00)	0.96 (0.94–0.98)	<0.001*	1.37 (1.34–1.40)	<0.001*
Civil servants	15 522 (27.00)	1.07 (1.05–1.10)	<0.001*	1.04 (1.02–1.07)	<0.001*
Immigrants	551 (1.00)	2.84 (2.59–3.11)	<0.001*	3.10 (2.81–3.42)	<0.001*
Other	9002 (16.00)	1.64 (1.59–1.68)	<0.001*	1.30 (1.27–1.34)	<0.001*
**Record status**					
Inpatient	55 140 (96.00)	ref		ref	
Short-time inpatient	87	0.05 (0.04–0.07)	<0.001*	0.08 (0.07–0.10)	<0.001*
Emergency department	2356 (4.00)	0.09 (0.09–0.10)	<0.001*	0.13 (0.12–0.13)	<0.001*
Unspecified	16	0.06 (0.04–0.10)	<0.001*	0.18 (0.11–0.29)	<0.001*

CI – confidence interval, OR – odds ratio, ref – reference

*Significant *P*-values (0.05).

**Figure 3:**
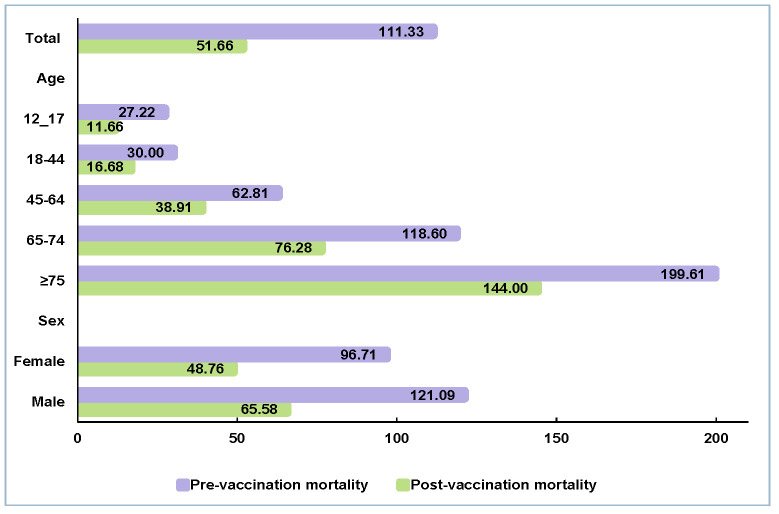
Comparison of COVID-19 in-hospital mortality risk per 1000 admissions per month between pre- and post-vaccination periods.

In-hospital mortality for COVID-19 before vaccination was 111.33 per 1000-monthly admissions. After 1% of Iranians received at least one dose of the COVID-19 vaccine, the mortality rate dropped to 51.66 per 1000 admissions per month. Mortality risk for those aged ≥75 years declined from 199.61 to 144.00. For the age group of 65–74 years, mortality risk was reduced from 118.60 to 76.28 per 1000 admissions per month. After immunisation, COVID-19 mortality risks declined in all other age groups. In the age groups of 45–64, 18–44, and 12–17 years, the COVID-19 in-hospital mortality risks were 62.81, 30.00, and 27.22 per 1000 admissions per month in pre-vaccination periods. Post-vaccination mortality risks declined to 38.91, 16.68, and 11.66. During the pandemic’s initial year, the in-hospital mortality risk for COVID-19 was 96.71 for females and 121.09 for males. However, post-vaccination these figures dropped to 48.76 for females and 65.58 for males (**Figure 3**).

**Figure 4:**
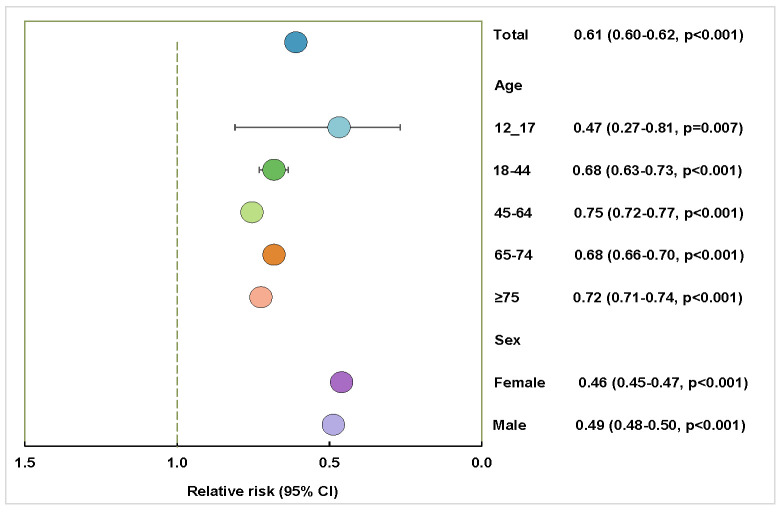
The relative risk of COVID-19 in-hospital mortality after vaccination compared to the pre-vaccination period as the baseline. All *P*-values are statistically significant (*P* < 0.05).

Post-vaccination, there was a noteworthy decrease in COVID-19 mortality (RR = 0.61; 95% CI = 0.60–0.62). In older age groups, the post-vaccination mortality risk reduced significantly for those aged ≥75 years and the age group of 65–74 years. For the age group of 45–64 years, the RR was 0.75 and for the age group of 18–44, the RR was 0.68. A decline in COVID-19 mortality risk was observed post-vaccination for both sexes (**Figure 4**).

## DISCUSSION

The first reported case of SARS-CoV-2 in Iran was confirmed in February 2020. Iranian authorities subsequently implemented lockdowns in response to COVID-19 surges in November 2020, September 2020, April 2021, and August 2021 [47,48]. Mask mandates and business activity restrictions varied depending on cities’ risk levels and businesses’ essential nature. In February 2021, Iran started its COVID-19 vaccination campaign. The Iranian population had access to various vaccines, including Sputnik V, Oxford/AstraZeneca, Covaxin, and Sinopharm. The inactivated vaccine was the most frequently administered type of vaccine in Iran. In prior studies in Iran, COVID-19 vaccines were found effective and safe. They showed reduced severe illness and mortality [32,34,35,41,49]. Mild side effects, such as local reactions, fatigue, chills, fever, muscle pain, headaches, and dizziness, typically resolve within 72 hours post-vaccination [19,34,40,41,50,51]. In this retrospective study, we examined the hospital admissions and mortality rates in Iran before and after COVID-19 vaccination across the population that received a variety of vaccine types.

The total data on COVID-19 hospital admissions underscored a heightened risk of hospitalisation with advancing age, a finding that aligns with several prior studies [14,52–57]. Additionally, females exhibited a greater rate of hospital admissions compared to males. Of the total 806 076 hospital admissions, 57 599 resulted in death. The death toll among those aged ≥75 years was the highest, with 25 560 deaths. The mortality risk of older age groups was significantly increased. An adjusted OR of 1.29 indicated that males faced a greater mortality threat from COVID-19 compared to females. Such observations resonate with previous studies, suggesting that advanced age and male sex correlate with higher mortality rates [56,58–62]. According to a meta-analysis of 28 studies, older adults had a higher mortality rate (OR = 7.86), and males had a higher mortality rate (OR = 1.82) among COVID-19, SARS, or Middle East respiratory syndrome (MERS) patients [63]. Although some studies have probed the correlation between chronic comorbidities and COVID-19 fatality rates, our study did not explore this topic.

### Hospital admissions

During the pre-vaccination phase in Iran, from 20 February 2020 to 20 April 2021, the COVID-19-related hospital admission rate was 459.78 per million population per month. This rate surged to 1178.66 during the post-vaccination phase from 21 April 2021 to 20 March 2022. Iran initiated its mass vaccination campaign on 9 February 2021, deploying the Sputnik V vaccine, followed by approvals for Oxford/AstraZeneca, Covaxin, Sinopharm and domestically produced vaccines were approved by the Ministry of Health and Medical Education. These vaccines encompassed varied mechanisms, with Covaxin and Sinopharm employing inactivated viruses, while Oxford/AstraZeneca and Sputnik V utilised non-replicating viral vectors. A significant portion of the Iranian population was inoculated with inactivated virus vaccines, with Sinopharm comprising approximately 83% of the total doses administered [30,31]. The other vaccines primarily consisted of AstraZeneca and Sputnik V, representing less than 9% of the total doses imported into Iran [31].

The overall IRR of the hospital admission rate during this period was 2.09 times the pre-vaccination rate. The surge in admission rates in this study correlates with the dominance of the Delta variant in Iran from 22 June 2021 to 22 September 2021 [64]. Initially identified in India in October 2020, the Delta variant has been a significant concern given its enhanced transmissibility and hospitalisation risks compared to the Alpha variant [65,66]. According to previous studies, the Delta variant was roughly 60% more transmissible than the Alpha variant due to several mutations in its spike protein [65,67–69]. While vaccines have shown slightly diminished efficacy against the Delta variant, they offer robust protection against severe illness and hospitalisation [65]. Prior research has emphasised the efficacy of COVID-19 vaccines in curbing severe cases necessitating hospitalisation even in the Delta variant, which contrasts with the findings of our study [2-14,17,20,21,32,57,70–72]. Research from Taiwan demonstrated the effectiveness of messenger ribonucleic acid (mRNA), protein subunit, and vector-based vaccines in preventing COVID-19 hospitalisation from 22 March 2021 to 30 September 2022, even in the presence of the Delta variant [73]. An observational study in Seychelles indicated that the Sinopharm vaccine maintained high levels of protection against hospitalisation, showing a 61% efficacy even in partially vaccinated individuals during the first wave of the pandemic driven by the Beta and Delta variants [74]. A cohort study in Thailand showed that vaccination reduced the risk of COVID-19 hospitalisation by 25% with a single vaccine shot [3]. However, the study highlighted the lower effectiveness of inactivated virus vaccines against the Delta variant than other vaccine types, particularly mRNA vaccines. A meta-analysis of vaccine effectiveness in over two million vaccinated individuals confirmed that inactivated vaccines had the lowest effectiveness against the Delta variant compared to other vaccine types [75].

In Chile, the estimated adjusted vaccine effectiveness for the inactivated SARS-CoV-2 vaccine in children aged 6–16 years was 91.0% for preventing hospitalisation during the Delta variant predominant period [2]. However, in our study, the hospital admission rate for children did not change significantly post-vaccination. A retrospective cohort study in China between May and June 2021, when the Delta variant was dominant, demonstrated the effectiveness of the inactivated vaccine in preventing B.1.617.2 variant SARS-CoV-2 infection [76]. The researchers emphasised the importance of full vaccination, as partial vaccination did not significantly alter outcomes compared to pre-vaccination [76]. Additional studies have indicated that a single dose of the inactivated vaccine exhibits limited effectiveness in preventing infection by the Delta variant [77,78].

The fifth COVID-19 wave in August 2021, dominated by the Delta variant, led to significantly higher hospitalisation and mortality rates, marking one of the most severe periods of the pandemic in Iran [79]. During the predominance of the Delta variant, a small percentage of individuals had been fully vaccinated, which may have contributed to severe symptoms, especially considering that the Delta variant was characterised by elevated levels of inflammatory markers such as C-reactive protein and erythrocyte sedimentation rate [36,80]. As the Delta variant began its spread in Iran, a mere 4.5% of the population had been vaccinated against SARS-CoV-2. Vaccination drives in developing nations often grapple with multifaceted challenges encompassing cultural, political, social, and economic dimensions [67,79,81]. Three months after the WHO emergency use approval for the Pfizer-BioNTech (BNT162b2) vaccine, Iran had received nearly 900 000 doses of COVID-19 vaccines [82,83].

Another plausible reason for the rise in COVID-19 hospital admissions could be tied to a diminishing adherence to health protocols designed to mitigate virus spread [79]. First, the protracted nature of the pandemic and the vaccination process have led to fatigue among the public, causing a decreased willingness to adhere to protective measures. Second, some individuals mistakenly believe they achieve full immunity immediately after receiving the first dose of the COVID-19 vaccine, leading them to relax their vigilance [79]. Third, rumours and concerns about vaccine side effects have sown distrust in certain population segments to postpone vaccination [79,82–85].

Another perspective on the increase in admissions during the pandemic’s second year, unrelated to vaccination, pertains to managing COVID-19 patients. This could be attributed to enhancements in patient management and resource allocation. During the early stages of the pandemic, many countries struggled with fostering inter-sectoral collaboration and integrated decision-making among policymakers and health care administrators, compounded by the inherent challenges of managing an unprecedented, rapidly evolving, and complex global health crisis [86–89]. At the pandemic’s outset, many hospitals grappled with infrastructural and resource constraints, including shortages in staff, equipment, and personal protective equipment [90–94]. However, as the pandemic evolved, decision-making processes underwent refinement, especially among members of Iran’s national COVID-19 committee. Consequently, there was a surge in hospital beds dedicated to COVID-19 patients, and the registration process for these patients improved due to increased polymerase chain reaction (PCR) testing capacities in Iran.

### In-hospital mortality

A significant finding from our study is the marked reduction in COVID-19 mortality risk post-vaccination. Before vaccination, the in-hospital mortality rate was 111.33 per 1000 admissions per month. This plummeted to 51.66 after at least 1% of the Iranian population received a vaccine dose. Notably, post-vaccination, all age groups witnessed decreased COVID-19 mortality risk. The vaccine’s introduction in Iran catalysed a substantial dip in COVID-19 mortality (RR = 0.61). Both sexes benefited from the vaccination, with a mortality relative risk of 0.46 for females and 0.49 for males. This stark reduction in mortality risk can also be attributed to the increased hospital admissions during the Delta variant surge, which naturally inflated the denominator. Post-vaccination, the older adult population experienced a decrease in mortality risk. In our study, the mortality rate decreased by 54% from the start of primary vaccination among health care staff and older adults. If the reductions in mortality rate were solely due to vaccination in Iran, the Delta surge mortality rate would have remained at 111.33 per 1000 admissions per month without vaccination. Consequently, an additional 32 141 COVID-19 patients died if vaccines were not available in a country.

A study in Iran’s Fars province found a significant COVID-19 mortality reduction (86–100%) among vaccinated individuals using various vaccine types [32]. In Gilan province, a study by Heidarzadeh et al. focused on the Sinopharm vaccine’s effectiveness. They found that the death OR within 1–30 days after receiving two doses of Sinopharm was 0.66, indicating mortality reduction [33]. Multiple studies confirm COVID-19 vaccinations’ effectiveness in reducing mortality, aligning with our observations across different countries and vaccine types [3,5,9-11,15,16,21,95]. A study by Nub et al. observed a notable decline in COVID-19 deaths, especially among priority vaccination groups, during the Delta variant surge [96]. Research by Vilches et al. emphasised the importance of accelerating the vaccination pace to achieve population-level immunity, pointing out that it would prevent many hospitalisations and deaths in the USA, even in states already impacted by the Delta variant [11]. McNamara highlighted that the initial roll-out of the USA COVID-19 vaccination program was associated with reductions in COVID-19 cases, ED visits, and hospital admissions, especially among older adults [17]. A study by Arregocés-Castillo et al. revealed a 79.8% vaccine efficacy in preventing mortality after COVID-19 hospitalisation among participants aged ≥60 years, accompanied by a substantial increase in hospital admissions caused by the Delta variant surge, leading to a rise in patient numbers [70].

### Study limitations

A limitation of our study is the unavailability of weekly data, which may reduce the accuracy of reported events. Additionally, due to the prolonged vaccination rollout in Iran, establishing a definitive timeline for vaccination is challenging. Vaccines were only available to health care workers in the initial phase, irrespective of age. Subsequently, vaccination was primarily age-based, without consideration of professional distinctions. For example, determining the precise vaccination date for the age group of 18–44 years was difficult because some individuals in this age group, who were health care workers, received their vaccines earlier than their peers.

Another limitation is that our study does not encompass all 1085 hospitals in Iran, as the IHIO has contracts with only 956 hospitals. IHIO members who sought medical care at non-contracted hospitals had to cover the costs personally or use secondary insurance, resulting in these hospital admissions not being recorded in the IHIO database and thus not included in this study.

Furthermore, the lack of access to outpatient data poses another limitation. Additionally, the insurance data are not linked with the vaccination database, preventing us from determining who received the vaccine and which type was administered. This disconnection hinders our ability to conduct a detailed analysis of vaccine-type effectiveness.

## CONCLUSIONS

In this study, we assessed the impact of COVID-19 vaccination on hospital admissions and mortality rates in Iran. Findings showed increased hospital admissions after vaccination due to the Delta variant but significantly reduced COVID-19 deaths. The vaccine effectively reduces severe outcomes, even against more transmissible variants. Post-vaccination, hospital admissions surged to 1178.66 per million population per month. However, mortality rates decreased from 111.33 to 51.66 per 1000 admissions per month. The RR for death post-vaccination was 0.61, indicating significant COVID-19 mortality reduction. These findings highlight the vaccine’s importance in curbing severe outcomes and mortality rates, particularly in older age groups. Future research should focus on longitudinal studies to explore the long-term impacts of COVID-19 vaccination on hospitalisation and mortality trends. This will provide deeper insights into the effectiveness of vaccine protection and inform more targeted public health strategies. Policymakers and health care authorities globally can benefit from these insights, reinforcing the importance of vaccination as a fundamental tool in controlling the pandemic and reducing severe disease outcomes.
